# Atlanta Medical College

**Published:** 1873-02

**Authors:** 


### ATLANTA MEDICAL COLLEGE.

From the delay in issuing this number of the JOURNAL, we are enabled to notice the commencement exercises of the above-named institution for the session of 1872–3, which came off on the evening of March 4th, at the Hall of Representatives, in the capitol building. Quite a large and appreciative audience assembled to witness the ceremonies, and, altogether, the occasion was one of more than usual interest.

The exercises were opened by an appropriate prayer by the Rev. Mr. Warren, of this city. Mr. John Hogan then delivered the valedictory address in behalf of the class. It is but justice to Mr. Hogan to say that he acquitted himself with honor, and reflected credit upon both the institution and the class he represented. His address was appropriate, chaste, eloquent, and delivered in an easy and graceful style.

The valedictory address in behalf of the faculty was delivered by Professor V. H. Taliaferro, in which some sound practical advice was given the graduating class.

Twenty-five graduates were then reported, and the degree of Doctor of Medicine conferred by Colonel W. L. Mitchell, of Athens, Georgia, preceded by an appropriate Latin address.

It appears, from the report of the Dean, that there have been in attendance as matriculates at the College, during the past session, ninety-three students. This report certainly shows the College in a highly encouraging condition.

The graduating class were remarkable for their genteel and intelligent appearance, and reflected credit upon their College.

Three prizes were awarded by the Professors of Surgery, Materia Medica, and Practice of Medicine, for best essays connected with their respective branches, viz: A fine pocket case of instruments to J. A. Beasley for the best essay on "The Improvements in Surgery for the last Twenty Years;" a medical case to L. O. McBride for the best essay on "Remedies;" a case of post-mortem instruments to C. M. Hill for the best essay on "Practical Medicine."

After the conclusion of the exercises, the students, faculty and trustees repaired to the Kimball House, where a sumptuous entertainment was prepared. The elite and fashion of the city were in attendance as invited guests, and, altogether, the occasion was one highly creditable and interesting.

